# A Combined Neutron and Proton Regimen for Advanced Salivary Tumors: Early Clinical Experience

**DOI:** 10.7759/cureus.14844

**Published:** 2021-05-04

**Authors:** Saif Aljabab, Andrew Lui, Tony Wong, Jay Liao, George Laramore, Upendra Parvathaneni

**Affiliations:** 1 Department of Radiation Oncology, King Saud University, Riyadh, SAU; 2 Department of Radiation Oncology, University of Washington School of Medicine, Seattle, USA; 3 Department of Medical Physics, Seattle Cancer Care Alliance Proton Therapy Center, Seattle, USA; 4 Department of Radiation Oncology, University of Washington, Seattle, USA

**Keywords:** salivary gland tumor, adenoid cystic carcinoma, neutron, proton

## Abstract

Background and objective

Fast neutron radiotherapy (NRT) is a high linear energy transfer (LET) particle therapy that offers a local control (LC) advantage over low-LET X-rays in the treatment of advanced and unresectable salivary gland malignancies. However, in tumors approximating the base of skull (BOS), target volumes may be underdosed to minimize toxicity to the central nervous system (CNS). In this setting, a proton beam boost to the underdosed part of the tumor may improve LC. We report our early experience with a hybrid neutron-proton approach in patients with BOS involvement.

Materials and methods

We retrospectively reviewed 29 patients with locally advanced and unresectable salivary gland tumors involving the BOS between 2014-2018. The median age of the patients was 56 years, with the majority of them having adenoid cystic carcinomas (ACC) (79%) with advanced T4a/b disease (86%), pathologic perineural invasion (PNI) (55.2%), and orbital invasion (34.5%). Five patients (17.2%) were cases of re-irradiation. Surgical resection was attempted in 15 patients (51.7%), of which none achieved negative margins. The median neutron dose was 18.4 neutron Gray (nGy) with a sequential proton boost (PB) with a median dose of 25 Gy [relative biological effectiveness (RBE)] (range: 16-45 Gy). Toxicity was graded per the Common Terminology Criteria for Adverse Events (CTCAE) version 4.03. Descriptive statistics and the Kaplan-Meier method were used.

Results

At a median follow-up of 18.9 months [interquartile range (IQR): 6.1-32.5], the entire cohort's overall survival (OS) was 93.1%, progression-free survival (PFS) was 79.3%, and LC was 89.7%. Among patients who were not re-irradiated (n=24), the most commonly recorded acute grade 3 toxicities were mucositis (50%) and dermatitis (37.5%). There was no documented acute grade 4/5 events. Late grade 3/4 events included trismus (n=1), hearing loss (n=2), visual loss (n=6), and bone necrosis (n=1). There were no reported late grade 5 events in de novo patients.

Conclusion

In this challenging cohort with a poor prognosis, early outcomes for a hybrid neutron-proton approach were found to be promising. Further studies involving longer follow-ups with a larger cohort of patients are required to validate our findings.

## Introduction

Salivary gland neoplasms are rare tumors with wide histological and anatomical diversity. They may arise in either major or minor salivary glands, involving structures such as the nasal cavity, paranasal sinuses, lacrimal gland, oral cavity, pharynx, larynx, trachea, and other sub-sites in the head and neck region. Aggressive salivary gland malignancies such as adenoid cystic carcinoma (ACC) and salivary duct carcinomas frequently involve the base of skull (BOS), either through direct extension or perineural invasion (PNI). The facial and trigeminal nerve pathways are commonly involved, which lie near critical structures such as the brain's temporal lobe, cerebellum, brain stem, optic structures, and cochlea. If left untreated, tumor progression may follow a relentless clinical course leading to significant morbidity (including neurological deficit, neuropathic pain, blindness, and hearing loss) or mortality. However, these tumors are notoriously difficult to treat due to their relative radioresistance and proximity to critical intracranial structures. Many are unresectable, and attempted surgical resections often turn out to be incomplete with gross residual disease and positive margins, leading to significant morbidity. Primary radiotherapy (RT) is usually the treatment of choice.

A phase III Radiation Therapy Oncology Group (RTOG)-Medical Research Council (MRC) trial compared conventional low linear energy transfer (LET) X-ray/electron radiotherapy (XRT) with fast neutron radiotherapy (NRT) and demonstrated the superiority of the latter for local control (LC) of tumor in advanced unresectable salivary gland malignancies. This study was stopped earlier than planned by the ethics review committee due to the highly significant improvement in the two-year local-regional control (LRC) of the NRT group vs. photon group (67% vs. 17%, p<0.005) and a trend toward improved survival (62% vs. 25%, p=0.1) [1]. With mature follow-up, the 10-year data showed an LRC of 56% vs. 17% (p=0.009), but there was no difference in overall survival (OS), which was driven by distant failures in both groups [2]. Neutrons appeared to alter failure patterns as more patients succumbed to distant metastasis rather than local failure. However, critical central nervous system (CNS) structures such as the temporal lobe of the brain, optic structures, and brain stem are more sensitive to neutrons' effects than non-CNS structures. To minimize the risk of neurological and vision complications, intentional underdosage in terms of the target volume close to or involving the BOS is necessary.

In a previous data analysis of patients treated with NRT alone at our center, we identified BOS invasion as a significant negative prognostic factor [3]. Patients without BOS invasion had a significantly better five-year LRC (70% vs. 19%, p<0.001). We postulated that the cause was underdosing to spare the CNS and optic structures. Around 2001, we implemented a strategy of boosting areas of relative underdosage in patients undergoing neutron therapy using Gamma Knife radiosurgery [4]. Compared to our historical experience with NRT alone, this combination improved LC in patients with BOS invasion, with rates approaching those of patients without BOS invasion. However, several patients would present with extensive intracranial invasion and large or anatomically complex tumor volumes that may not be optimal for Gamma Knife radiosurgical boost. Hence, there was a need to explore additional strategies to improve LC.

Our proton therapy center became operational in 2013, and pencil beam scanning was commissioned in late 2013. In 2014, we started using a fractionated treatment course with proton therapy as a boost following NRT in patients with BOS-invasive salivary gland tumors that were large or irregular and felt to be poorly suited for a Gamma Knife boost. This retrospective study analyzes the initial clinical outcomes of this novel combination particle beam approach: NRT followed by a proton boost (PB). We hypothesized that in a challenging cohort of patients with BOS invasion, a hybrid course would be feasible with minimal additional toxicity and optimized LC. This study presents the early clinical and toxicity-related results of this novel approach.

## Materials and methods

Study design, endpoints, and ethics approval

We retrospectively reviewed patients who were treated at our center from 2014 to 2018 with NRT followed by a PB. This study included patients with a pathologically confirmed diagnosis of a locally advanced salivary gland malignancy that received definitive, postoperative, or salvage RT. We included patients who had prior RT, and their toxicity outcomes were reported separately. The primary endpoint was LC. Secondary endpoints included progression-free survival (PFS), OS, and toxicity outcomes. The study protocol was reviewed and approved by our local Institutional Review Board (IRB) committee.

Evaluation, treatment, and data collection

All patients were evaluated by our head and neck multidisciplinary tumor conference, which includes head and neck surgeons, medical oncologists, radiation oncologists, and neuroradiology. Unresectable cases were generally referred for definitive radiation. Patients deemed resectable underwent surgical resection and then received postoperative RT when indicated. Generally, postoperative RT indications included a positive margin (R1), gross residual disease (R2), positive PNI, multiple positive nodes, extra-nodal extension, and ACC or other high-grade histologies. Before initiating RT, patients were evaluated by our dental team for pre-NRT clearance and, if applicable, a custom oral stent was developed. Standard immobilization with a thermoplastic mask was used at simulation.

**Figure 1:**
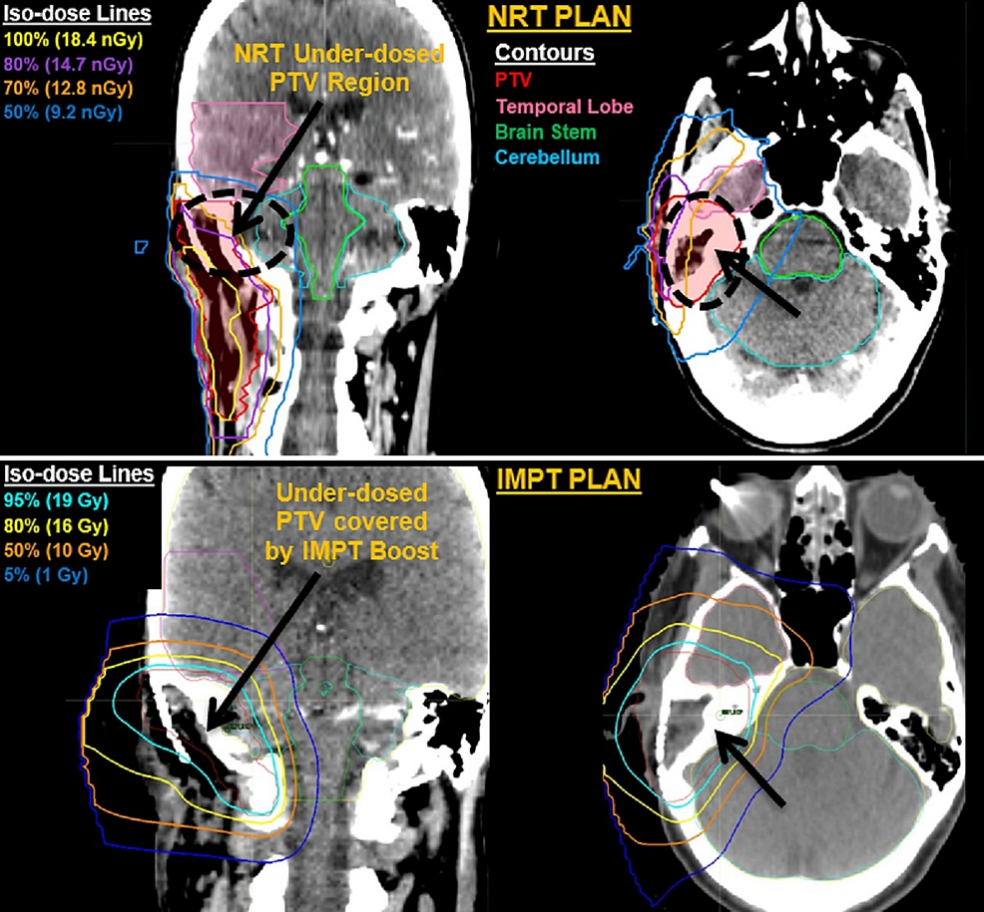
Images of a patient treated with neutron therapy followed by an IMPT The images are of a 64-year-old male patient presenting with an adenoid cystic carcinoma (T4a) status-post gross total resection with positive margins (R1) and pathologic perineural invasion. He underwent postoperative neutron radiotherapy (top) with a dose prescription of 18.4 nGy in 16 fractions followed by a proton boost (IMPT, bottom) with a dose prescription of 20 Gy (RBE) in 10 fractions to the underdosed PTV region Contours: PTV (red), temporal lobe (pink), brain stem (green), and cerebellum (light blue). (Top) NRT plan isodose line (dose, color): 100% (18.4 nGy, yellow), 80% (14.7 nGy, purple), 70% (12.8nGy, orange), 50% (9.2, navy blue). (Bottom) IMPT plan isodose line: 95% (19 Gy, cyan), 80% (16 Gy, yellow), 50% (10 Gy, orange), 5% (1 Gy, navy blue) IMPT: intensity-modulated proton therapy; RBE: relative biological effectiveness; PTV: planning target volume; NRT: neutron radiotherapy

Neutron plans were delivered with a high-energy, hospital-based Scanditronix MC™ 50 cyclotron. The cyclotron utilizes a 50.5 MeV p→Be reaction and is equipped with an isocentric rotating gantry and multi-leaf collimation system, allowing conformal field shaping. NRT treatment planning was performed using the Pinnacle 3 (version 9.0) planning system on a contrast CT simulation scan. Diagnostic MRI imaging was often used to assist in tumor volume delineation. The dosimetric characteristics of the NRT system have been previously described [5]. Treatment fields were individualized according to the primary tumor’s location and extent, typically with a forward planned 3D conformal approach using two to five fields. The temporal lobe dose was limited to approximately 13.5 neutron Gray (nGy), brain stem to 12 nGy, and optic nerves and chiasm to 11.5 nGy. The median neutron dose to the gross disease and postoperative high-risk volume was 18.4 nGy (range: 9.2-18.4 nGy) in 16 fractions (range: 8-16 fractions) at 1.15 nGy per fraction delivered four times per week over four weeks. Towards the third or fourth week of treatment, patients who required a PB underwent repeat CT simulation at the proton center with a reproduction of the initial patient setup and neck extension confirmed with image fusion in MIM (MIM Software Inc., Cleveland, OH). All PB plans were delivered sequentially after the completion of neutron therapy. Plans with one to three beams were generated using a pencil beam dose calculation algorithm in 21 patients, and for the more complex targets (eight patients), the Monte-Carlo dose calculation algorithm was used. Most plans (25 patients, 86.2%) were treated using pencil-beam scanning technique, and uniform scanning was only used in four patients (13.8%). Proton radiation fields were limited to the underdosed regions from the neutron therapy plan. This region was determined by fusing the neutron plan with the proton therapy simulation scan and delineating the residual disease underdosed in the neutron phase. Typically, this included tumor volumes that received only 10-16 nGy, rather than the full dose of 18.4 nGy. The underdosage degree determined the PB dose after adjusting for a neutron relative biological effectiveness (RBE) of 7-8 for the tumor and 4-4.5 for the CNS or optic structures, and all these cases were peer-reviewed by our head and neck radiation oncology group. Representative images of a patient treated with neutron therapy followed by intensity-modulated proton therapy (IMPT) boost are illustrated in Figure 1.

All patients receiving a PB were enrolled in a multicenter IRB-approved national registry. Patients were evaluated every week during the neutron and proton radiation. Post-treatment evaluations were performed every three to six months during the first year, every four to six months during the second year, every 6-12 months during the period covering third to fifth years, and annually after that. Follow-up imaging included a CT scan at three months and an MRI at six months. Both provide the new baseline for future response evaluation. Thereafter, patients with high-grade tumors will receive a CT scan every three to four months for two years, and then every six months for two additional years, and then annually. Patients with low-grade tumors, such as adenoid cystic and acinic cell cancers, will receive a CT scan every six months until five years, and then annually. Further MRI imaging is only required when suspicious findings are detected on follow-up CT imaging. Post-treatment evaluations were performed either by our center head and neck team or by the patient’s local medical team for out-of-state and out-of-country patients (n=15, 51.7%). The follow-up images were reviewed at our center, and patients were contacted virtually for any additional information or necessary clarifications. Toxicity was assessed and recorded according to the Common Terminology Criteria for Adverse Events (CTCAE) version 4.03 [6]. Acute toxicity was defined as that which occurred in the period from the start of RT till three months post-treatment, whereas late toxicities were defined as those which occurred in the period from three months post-RT and onwards.

Statistical analysis

Descriptive statistics were used to characterize the baseline characteristics of the overall population. We defined LC as stable disease with no further tumor growth following RT. Survival times were computed from the RT end date to the occurrence of the first event. Events were death from any cause for OS and any recurrence or death for PFS. Patients were censored at the last follow-up visit or after a recorded event. Survival rates were estimated using the Kaplan-Meier method. Analyses were performed with IBM® SPSS® Statistics version 25 (IBM, Armonk, NY).

## Results

Patient and tumor characteristics

A total of 29 patients with a median age of 56 years were treated for locally advanced salivary gland tumors at our center from March 2014 to March 2018. The patient and tumor characteristics are listed in Table 1. All patients had a pre-treatment Eastern Cooperative Oncology Group (ECOG) performance status of 0-2. The majority of patients were Caucasian males (n=22, 76%) with T4a/b disease (n=25, 86.2%) and ACC histology (n=23, 79%). One patient had a large bulky synovial sarcoma that had invaded the skull base but was deemed T2 per sarcoma staging guidelines. The remaining three patients had bulky T3 disease with R1/R2 resections. The orbital invasion was seen in 10 patients (34.5%) and pathologic PNI was detected in 16 patients (55.2%).

**Table 1:** Patient and tumor characteristics *These three patients had in-field recurrences in previously irradiated fields. The treated recurrence involved the skull base including the cavernous sinus along the pathway of the V2 division of the trigeminal nerve and the orbits. The original primary for two of these cases was the paranasal sinuses and hard palate in one BOS: base of skull; IQR: interquartile range

Variables		Values	
Median age (years), IQR		56	47-64
Characteristic		N	%
Gender			
Male		18	62%
Female		11	38%
Ethnicity/race			
Caucasian		22	76%
Site			
Paranasal		9	31%
Palate		5	17.20%
Parotid		5	17.20%
Nasal		3	10.30%
Nasopharynx		2	6.90%
External auditory canal		1	3.50%
Oral cavity		1	3.50%
Skull base*		3	10.30%
Histology			
Adenoid cystic carcinoma		23	79%
Adenocarcinoma		2	6.90%
Sino-nasal carcinoma		2	6.90%
Synovial sarcoma		1	3.50%
Acinic cell carcinoma		1	3.50%
Disease Extension			
T4a/b		25	86.20%
Node-positive		1	3.50%
Intra-cranial extension		9	31%
Orbital invasion		10	34.50%
Pathologic perineural invasion		16	55.20%

Treatment characteristics

The treatment characteristics are outlined in Table 2. A total of 21 patients had de novo disease, and eight patients had a recurrent disease, of which five had received prior radiation. Before initiating neutron therapy, surgical resection was attempted in 15 patients (51.7%). Eight patients achieved a gross total resection with positive margins (R1), and seven had a sub-total resection with residual disease (R2). The remainder of the patients (48.3%) had unresectable gross disease requiring definitive RT. One patient with synovial sarcoma (T2) received additional six cycles of neoadjuvant chemotherapy (ifosfamide, Adriamycin, and mesna) prior to achieving a gross total resection with positive margins (R1). The median neutron dose was 18.4 nGy (range: 9.2-18.4 nGy) in 8-16 fractions at 1.15 nGy per fraction four times a week. The median PB dose was 25 Gy (RBE) (range: 16-45 Gy) in 10 fractions (range: 8-20 fractions) at a median dose per fraction of 2 Gy (RBE) (range: 1.8-2.66 Gy). The median time from NRT initiation to PB end was 43 days [interquartile range (IQR): 40-50].

**Table 2:** Treatment characteristics RT: radiotherapy; RBE: relative biological effectiveness

Characteristics		N	%
Surgery			
Total Pre-RT resections		15	51.70%
R0 resection		0	0%
R1 resection		8	27.60%
R2 resection		6	20.70%
Unknown margin status		1	3.40%
Biopsy only		14	48.30%
Chemotherapy			
Neoadjuvant chemotherapy		1	3.50%
No chemotherapy		28	96.60%
Radiotherapy			
Definitive RT		21	72.40%
Salvage RT		3	10.30%
Salvage re-irradiation		5	17.20%
Neutron dose (nGy), median (range)		18.4	9.2-18.4
Proton dose (Gy, RBE), median (range)		25	16-45

Re-irradiation patient characteristics

A total of five (17.2%) re-irradiation cases were included in this analysis. All had a prior diagnosis of ACC with prior radiation ≥10 years ago. Two received neutron therapy (18 nGy and 19.2 nGy respectively), and three received photon therapy (two received 59.4 Gy, and one received 70 Gy). All had a recurrence of T4a/T4b ACC disease involving the skull base, of which only two had an attempted resection. One was a gross total resection with positive margins and positive PNI. The other was a sub-total resection with gross residual disease. All five cases proceeded with neutron re-irradiation with a median neutron dose of 13.8 nGy, and a PB median dose of 30 Gy.

Survival outcomes

**Figure 2:**
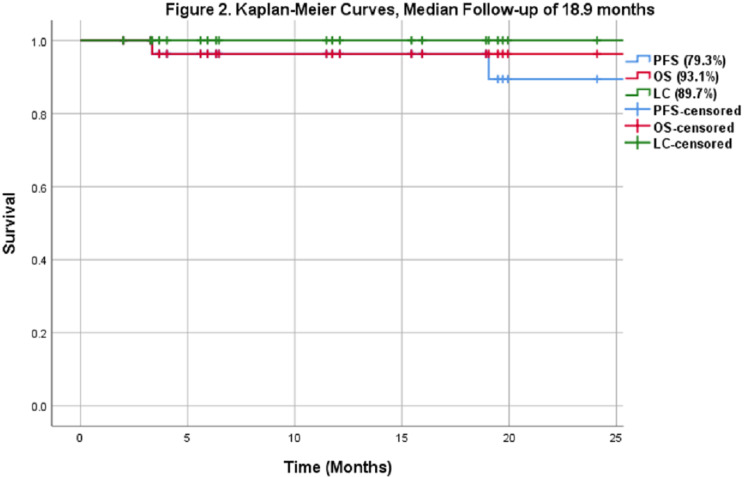
Kaplan-Meier curves (median follow-up of 18.9 months) PFS: progression-free survival; OS: overall survival; LC: local control

At a median follow-up time of 18.9 months (IQR: 6.1-32.5), the entire cohort’s local control was 89.7%, PFS was 79.3%, and OS was 93.1% (Figure 2). The OS and LC for the de novo sub-group that received definitive RT (n=21) were 95.2% and 85.7%, respectively. We identified three local failures in this sub-group, which were determined through imaging, and only one case was pathologically confirmed with a biopsy. None of the recurrences were localized to the proton/neutron match region. Two of the three local recurrences received salvage chemotherapy and are still alive. The third patient did not receive any salvage therapy and passed away several months later. One additional patient in this sub-group developed distant recurrence only and is still alive. In the salvage RT and re-irradiation subgroup (n=8), OS and LC were 87.5% and 100%, respectively. One patient developed a distal recurrence but is still alive. Another patient that received re-irradiation developed intracranial MRI changes consistent with radiation necrosis five months post-treatment and soon passed away.

Toxicity

In general, acute toxicity was very tolerable, and late toxicities were acceptable, with most patients experiencing toxicity from the NRT phase. Toxicity rates for all de novo irradiated cases (n=24) are listed in Table 3. Excluding re-irradiated patients, a total of 15 patients (62.5%) developed acute grade ≥3 events. Commonly recorded acute grade 3 toxicities were mucositis (50%) and dermatitis (37.5%). Two patients (6.9%) required hospital admission and percutaneous endoscopic gastrostomy (PEG) tube insertion due to decreased PO intake and for pain control. Eight patients (33.3%) developed in-field infections that resolved with antibiotic therapy. No patient experienced a grade 4 or 5 acute event.

Regarding late adverse events, a total of seven patients (29.2%) developed grade ≥3 adverse events, with three patients developing multiple grade ≥3 events. Late events included trismus in one patient (grade 3), hearing loss in two patients (grade 4), and anopsia in six patients (grade 4). All patients that developed anopsia had either orbital tumor involvement or orbital approximation at presentation. Two had presented with proptosis, and one patient had presented with eye pain and diplopia. Post-treatment, two patients experienced unilateral visual loss at three to six months (one vitreous hemorrhage and one retinopathy), and four patients experienced unilateral visual loss at 24-36 months (two glaucoma cases, one corneal ulceration, and one retinopathy). Patients that developed anopsia had no tumor recurrence or local disease progression. Brain necrosis was recorded in two patients, and both were asymptomatic brain necrosis in the temporal lobe identified on routine MRI imaging, and no therapy was required for those (grade 1). Osteoradionecrosis was recorded in two patients. Both occurred in the postoperative radiation setting; one was a grade 2 event, and the other was a grade 4 event requiring surgical debridement. There were no recorded grade 5 events in the de novo setting.

All patients in the re-irradiated sub-group (n=5) developed grade 1-3 acute toxicities with no recorded grade ≥4 acute adverse events. They all completed their re-irradiation course without requiring a PEG tube insertion or hospitalization. One patient developed an in-field infection requiring antibiotic therapy. Late grade ≥3 events included unilateral visual loss (grade 4) in two patients, unilateral hearing loss (grade 3-4) in two patients, trismus (grade 3) in one patient, and bone necrosis (grade 3) in one patient requiring hyperbaric oxygen therapy. One patient who had received prior NRT developed a possible (grade 5) brain necrosis event as previously detailed.

**Table 3:** Peak acute and late radiation toxicity grade recorded among the 24 cases who received de novo irradiation, per the CTCAE version 4.03** *Expected events secondary to disease extension or approximation into the orbit; **excluded re-irradiation cases (n=5) CNS: central nervous system; CTCAE: Common Terminology Criteria for Adverse Events

Toxicity	Grade 1-2		Grade 3		Grade 4		Grade 5	
	N	%	N	%	N	%	N	%
Acute								
Dermatitis	15	62.50%	9	37.50%	0	0%	0	0%
Pain	20	83.30%	2	8.30%	0	0%	0	0%
Mucositis	11	45.80%	12	50%	0	0%	0	0%
Weight loss	12	50%	0	0%	0	0%	0	0%
Xerostomia	22	91.60%	0	0%	0	0%	0	0%
Dysgeusia	19	79.20%	0	0%	0	0%	0	0%
Late								
Dysphagia	2	8.30%	0	0%	0	0%	0	0%
Xerostomia	15	62.50%	0	0%	0	0%	0	0%
Visual loss	0	0%	0	0%	6*	25%	0	0%
Hearing loss	7	29.20%	0	0%	2	8.30%	0	0%
Trismus	3	12.50%	1	4.20%	0	0%	0	0%
CNS stroke	1	4.20%	1	4.20%	0	0%	0	0%
Bone necrosis	1	4.20%	0	0%	1	4.20%	0	0%
Brain necrosis	2	8.30%	0	0%	0	0%	0	0%

## Discussion

Radiation therapy is a crucial modality for managing locally advanced unresectable or incompletely resected salivary gland tumors, especially ACC and other high-grade histologies. Malignant salivary gland tumors with skull base invasion present significant therapeutic challenges. Although surgical resection is associated with the best outcomes, it is often not feasible due to very high rates of morbidity associated with it. In addition, systemic therapy is ineffective at addressing both local and distant diseases, and these tumors are recognized as relatively radioresistant [7]. Finally, the proximity of the tumor to critical structures limits the ability to deliver RT safely. LC is vital in this setting as uncontrolled tumor progression can lead to substantial morbidity and severe detriment to the patient’s quality of life secondary to neurological complications such as vision loss, hearing loss, cranial nerve deficits, and neuropathic pain.

Another challenge is to minimize treatment-related morbidity. Although NRT is an effective option for locally advanced salivary gland malignancies, delivering a full dose is not feasible in patients with significant skull base invasion or intracranial extension [3]. The intracranial and optic structures most often pose dose-limiting constraints when treating this highly complex BOS region to control a relatively radiation-resistant histology [8]. To limit the unacceptable levels of morbidity, the sub-volume of the tumor near the critical structure is intentionally underdosed, which has been shown to decrease LC rates.

We have previously reported on using NRT with a Gamma Knife boost, which improved LC compared to neutron therapy alone and with acceptable toxicity. We recently presented an update of this experience showing favorable LC outcomes [4,9]. However, Gamma Knife radiosurgery may not be suitable for large intracranial/skull base targets or irregular volumes, such as those seen when targeting cranial nerve PNI pathways. In addition, the delivery of a single large radiation fraction (9-12 Gy) after a course of NRT may have a higher risk of temporal lobe necrosis compared to a more fractionated approach. In this study, we explored the use of NRT with a fractionated PB to further reduce treatment-related toxicities.

To our knowledge, this is the first paper to report clinical experience related to the use of neutron therapy in combination with a proton beam boost. Prior studies have established the radiobiologic effectiveness of high-LET particle therapies such as neutrons and carbon ions compared to low-LET photons [2,10,11]; we outline these differences in Table 4. Our early results have shown promising short-term outcomes in a cohort of patients with challenging locally advanced disease. Nearly a third of patients in this cohort had recurrent disease. Although the number of evaluated patients was small and with a short median follow-up of 18.9 months, the LC achieved was promising at 89.7%. However, longer follow-ups are required as late failures are known to occur, especially in ACC.

**Table 4:** RBE for neuronal and non-neuronal normal tissues using photons, protons, neutron, and carbon ions The above applies for a standard fractionation of 2 Gy per fraction for photon and proton-based radiotherapy and 1.15 Gy per fraction for neutrons and carbon ions. Non-neuronal normal tissues include structures such as the bone, soft tissues, cartilage, and skin. Neuronal tissues include structures such as the brainstem, spinal cord, and brain RBE: relative biological effectiveness

RBE	Photon	Proton	Neutron	Carbon ion
Non-neuronal normal structures	1	1.1	3-3.5	3-3.5
Neuronal tissues	1	1.1	4-4.5	4-4.5
Salivary gland tumors	1	1.1	7-8	7-8

Although comparing outcomes between retrospective series is not very fruitful and should be done with caution, our results are consistent with previous particle therapy studies that included locally advanced salivary gland tumors with skull base invasion. We have previously published our experience with NRT alone as well as that with neutron plus Gamma Knife boost experience [3,4,9]. We reported a 24-month LC rate of 81% (NRT alone) vs. 82% (NRT plus Gamma Knife). The difference widened significantly (p=0.04) at 40 months with an LC of 39% vs. 82%. The late toxicity rate (grade ≥3) in the latter arm was 22.8%, and only three patients developed grade 1-2 neurologic adverse events, which is comparable to our results. Jensen et al. reported on the German experience using raster-scanned carbon ion therapy plus intensity-modulated radiotherapy (IMRT) [11]. They reported a three-year LC rate of 83.7% and a late toxicity rate (grade ≥3­) of <5%. It is important to note that their series included only a few patients with BOS involvement (<10%). Sulaiman et al. reported on the Japanese experience using high-LET carbon ion radiation alone [12]. They reported a two-year LC rate of 88% and a late toxicity rate (grade ≥3) of 15%. They also reported two grade 5 events. Although our early outcomes are consistent with some proton therapy-alone series [13,14], Pommier et al. reported a much higher LC rate of 100% at two years and 93% at five years using a dose-escalated mixed proton and photon beam approach [15]. However, they reported a higher number of neurologic grade ≥3 events (12 of 23 patients), of which two were grade 5 events. Only a few cohorts have reported the use of photon RT alone in the setting of locally advanced salivary gland tumors. Münter et al. reported a three-year LC rate of only 38% using IMRT alone and concluded that it was a feasible treatment for ACC if particle therapy was not available [16]. Unfortunately, only a few photon-alone series reports on late toxicity rates exist, and most of these series involve the early-stage and resectable settings [7,17-20].

Acute toxicity in our study was very tolerable, with most patients developing expected in-field mucositis and dermatitis. The pattern of acute symptoms was unique in that it usually peaked at the end of the neutron phase and subsided by the end of the PB phase. The median time between both phases was three days (range: 1-24 days). Nearly a third of our cohort had developed a facial infection several weeks post-treatment (i.e., facial cellulitis). Most of them resolved with intravenous antibiotics, and only one required surgical debridement with flap reconstruction. Many of these patients presented in the postoperative setting, which may have elevated the risk of infection with RT.

Regarding late adverse events, we recorded one grade 5 event in a neutron re-irradiation case. The changes included a complex contrast-enhancing lesion in the temporal lobe within the RT field on the T1-weighted scan and white matter high signal edema on the T2/Flair study. Perfusion sequences did not show increased relative cerebral blood flow in the index lesion, further supporting the diagnosis of radiation injury. Given the lack of biopsy findings, we cannot exclude a recurrence. However, the event’s timing and the radiological changes made us favor a diagnosis of a treatment-related brain necrosis case (grade 5 event).

Excluding re-irradiation cases (n=24), nearly a third of our patients (n=7, 29.2%) developed grade ≥3 adverse events with no recorded grade 5 events. In this sub-group, the incidence of radiation-induced brain injury with this hybrid modality was low, with only two patients developing asymptomatic brain injury (grade 1). We report a higher risk of grade 4 unilateral anopsia compared to other studies [3,11,12,14,15]. In these previously unselected series, anopsia events ranged from 0.1 to 4.3%, while in our series, it was considerably higher at 25%. We attribute this higher risk to the more advanced nature of the disease in our cohort, with nearly a third of our patients presenting with orbital involvement. Half of our anopsia events occurred in patients who had visual impairment prior to the treatment. The visual loss was unavoidable in the remaining patients due to disease approximation to the orbit. In these cases, a decrease in coverage would potentially increase the risk of local recurrence.

Despite our efforts, there are several limitations to our study. Firstly, as a retrospective study, it is subject to statistical bias, such as attrition, information, and selection bias. Secondly, nearly half of our patients were out-of-state or international referrals and this resulted in limited access to certain follow-up and imaging reports. We mitigated this by contacting patients directly through phone interviews and remote access to regional electronic health records. Thirdly, our study was based on a small cohort of patients. This was unavoidable as we were evaluating a highly select group of patients with a rare disease site. Finally, the described treatment's technical complexity limits its reproducibility at other facilities that wish to explore a similar approach with high-LET substitutes. Carbon ion radiation with PB may be a potentially superior approach. However, facilities with carbon ion capabilities are also very rare.

## Conclusions

Malignant salivary gland tumors with skull base invasion and perineural spread are challenging to treat. A novel combined neutron and PB treatment approach is tolerable with encouraging short-term LC rates. Studies that involve longer follow-ups with a larger cohort of patients are necessary to confirm these early results. Distant failure remains a concern, and improvements in systemic therapy strategies are needed. We are currently planning to launch a clinical trial that combines neutron therapy with immunotherapy in this setting.
